# Perioperative management of caesarean section for a pregnant woman with Sjögren's disease and pulmonary embolism: a case report

**DOI:** 10.3389/fcvm.2025.1467309

**Published:** 2025-04-11

**Authors:** Fanghao Liu, Xiaohui Wang, Shu Yuan, Lin Xu, Kaiyue Shan, Longfei Wang, Jianting Huang, Qiang Zheng

**Affiliations:** Department of Anesthesiology, Qilu Hospital (Qingdao), Cheeloo College of Medicine, Shandong University, Qingdao, China

**Keywords:** Sjögren's disease, autoimmune diseases, pulmonary embolism, venous thromboembolism, pregnancy, female, cesarean section, case report

## Abstract

**Background:**

Sjögren's disease (SjD) is a chronic inflammatory autoimmune disease with significant female predominance, characterised by lymphocyte proliferation and progressive damage to exocrine glands. The complexity of the condition of women with SjD and the incidence of complications substantially increase during pregnancy, which undoubtedly has consequences on both maternal health and neonatal outcomes. Pulmonary embolism (PE) is associated with increased perinatal mortality. However, PE has rarely been reported in pregnant women with SjD.

**Case presentation:**

A 40-year-old pregnant woman was diagnosed as having SjD. During admission, she experienced chest tightness and suffocation and was scheduled for caesarean section under combined spinal-epidural anaesthesia because of sustained low oxygen saturation and foetal distress. Postoperative pulmonary artery computed tomography angiography confirmed that the patient had developed a pulmonary embolism during the perioperative period. After multidisciplinary consultation, the patient underwent inferior vena cava filter implantation, anticoagulation, oxygen therapy, and anti-infection therapy; both the mother and neonate recovered and were discharged.

**Conclusion:**

Early identification and comprehensive perioperative monitoring during the prenatal period are vital in patients with SjD complicated by PE.

## Introduction

Sjögren's disease (SjD) is a complex chronic inflammatory autoimmune disease characterised by reduced secretion of exocrine glands, which predominantly damages the salivary and lacrimal glands, nose, upper respiratory tract, and oropharynx; the vagina may also be affected. The pathogenesis of SjD is multifaceted and involves both genetic predispositions and environmental triggers (1). It is characterised by a chronic inflammatory process within the exocrine glands, driven by an abnormal immune response to autoantigens, such as Ro/SSA and La/SSB, mediated by both B and T cells. This immune dysregulation results in the infiltration of lymphocytes into exocrine glands, causing damage to their physiological structure and function (2).

The reported incidence and prevalence of SjD differ according to the classification criteria applied. A comprehensive systematic review of relevant studies estimated the incidence at approximately 6.9 cases per 100,000 person-years and prevalence of approximately 60.8 cases per 100,000 individuals (3). SjD predominantly affects females, with a female-to-male ratio of 9:1–28:1 (4). The prevalence of secondary SjD, which occurs in conjunction with other autoimmune diseases, fluctuates according to the specific associated conditions.

SjD is a slowly progressive condition with a wide range of clinical manifestations. Patients often present with sicca symptoms, including dry eyes and dry mouth, but may also experience extraglandular involvement, such as arthritis, neuropathy, pulmonary disease, and renal complications (5, 6). This disease has various clinical characteristics and induces multiple systemic complications that cause difficulties and challenges in perioperative anaesthesia management. Research has shown that SjD can promote the development of a hypercoagulable state of blood, thereby increasing the risk of venous thromboembolism (VTE) and pulmonary embolism (PE). This hypercoagulability is thought to be related to the chronic inflammatory state and immune dysregulation characteristic of SjD (7). In addition, the risk of adverse pregnancy outcomes increases in women with SjD, underscoring the need for specialised care during pregnancy and the perinatal period (8).

We report the case of a 40-year-old female patient with SjD who underwent caesarean section and developed PE during the perioperative period. Here, we discuss our management strategies for this case, emphasising the need for vigilant monitoring, appropriate thromboprophylaxis, and careful consideration of the unique challenges posed by SjD in the perioperative setting.

## Case presentation

A 40-year-old woman at 37 weeks of pregnancy complained of chest tightness and suffocation and visited our hospital for treatment on 4 April 2024. This patient was previously diagnosed with SjD at another hospital, after which she received treatment with oral hydroxychloroquine sulphate (0.2 g twice a day) for 5 weeks until admission. Symptoms on admission included limb fatigue, palpitations, dry mouth, dry eyes, and Raynaud phenomenon. The patient's vital signs were recorded upon admission. The patient's non-invasive blood pressure was 120/75 mmHg, heart rate fluctuated between 110 and 130 beats per minute, and respiratory rate and body temperature were 15 beats per minute and 36.5°C, respectively. Analyses of the results of the autoantibody and routine examinations were as follows: the anti-SSA antibody concentration was 67.62 RU/ml, the antinuclear antibody (ANA) was positive, the anti Ro52 antibody concentration was greater than 400 RU/ml; complete blood count revealed that the white blood cell count was 9.03 × 10^9^/L, haemoglobin concentration was 136 g/L, and platelet count was 214 × 10^9^/L; plasma D-dimer concentration was 15.97 μg/ml, B-type natriuretic peptide concentration was 200 pg/ml; sensitive troponin I concentration was 0.624 ng/ml; and creatine kinase isoenzyme concentration was 7.51 ng/ml. No apparent abnormalities were found in the other laboratory test results (immunological test results are shown in Table 1). However, foetal heart monitoring revealed that although it manifested as regular uterine contractions, there were frequent deceleration phenomena, and the lowest foetal heart rate could be reduced to 86 beats/minute. Based on the patient's symptoms and results of clinical examinations, as well as consultation opinions from relevant departments, surgeons considered foetal distress and suggested emergency caesarean section to deliver the pregnancy.

**Table 1:** Preoperative rheumatoid immune-related test results.

Project Name	Test Results	Concentration Unit	Reference Range
Anti-nRNP/Sm antibody	<2.00	RU/ml	0–20
Anti-Sm antibody	3.14	RU/ml	0–20
Anti-SSA antibody	438.52	RU/ml	0–20
Anti-SS-B antibody	<2.00	RU/ml	0–20
Anti-Scl-70 antibody	<2.00	RU/ml	0–20
Anti-Jo-1 antibody	<2.00	RU/ml	0–20
Anti-AMA-M_2_ antibody	3.37	RU/ml	0–20
Anti-GBM antibody	<2.00	RU/ml	0–20
Anti-PR3 antibody	<2.00	RU/ml	0–20
Anti-MPO antibody	<2.00	RU/ml	0–20
Anti-dsDNA antibody	<2.00	IU/ml	0–30
Anti-ACA antibody lgG/lgA/lgM	3.48	RU/ml	0–20
Anti-ANA antibody	Positive		Negative
Main karyotype	Homogeneous		
Main karyotype titres	1:640		
Secondary karyotype	Granular		
ASO	<51.90	IU/ml	0–200
RF	14.30	IU/ml	0–20

The patient was urgently transferred to the operating room 2 h after admission, and a routinely established vein circuit was used to monitor the non-invasive arterial pressure, heart rate, electrocardiography, and pulse oxygen saturation. The patient's blood oxygen saturation was 90%–91% without oxygen inhalation and increased to 97%–98% after mask oxygen inhalation. Arterial blood gas analysis was performed immediately; the lactate value was 1.9 mmol/L, end-tidal carbon dioxide (ETCO_2_) pressure was 25 mmHg, and other parameters were generally normal. Considering the abovementioned symptoms and laboratory results, PE was considered. The possibility of acute aortic dissection, coronary syndrome, and left heart failure pulmonary oedema persisted. Subsequently, the patient was manoeuvred into the appropriate position, and the intervertebral lacunae were selected between L3 and L4 as puncture points to implement combined spinal-epidural anaesthesia with 0.5% ropivacaine 2.5 ml. When the sensory block reached T6, the surgeon commenced the surgery. A 3.15 kg female infant was delivered using a transverse incision in the lower abdomen, with an Apgar score of 10 points at 1 and 5 min after delivery. Subsequently, 4 ml of 1.5% lidocaine was administered via the epidural catheter, and 20 mg of furosemide was administered via intravenous injection to reduce the volume load. The course of anaesthesia and surgery was uneventful, and the patient received 500 ml of physiological saline. The urine volume and blood loss were 150 and 300 ml, respectively, during the entire surgery. The patient returned to the ward after the removal of the epidural catheter and connection of patient-controlled intravenous analgesia (PCIA) with sufentanyl and dexmedetomidine at the end of the operation.

**Figure 1:**
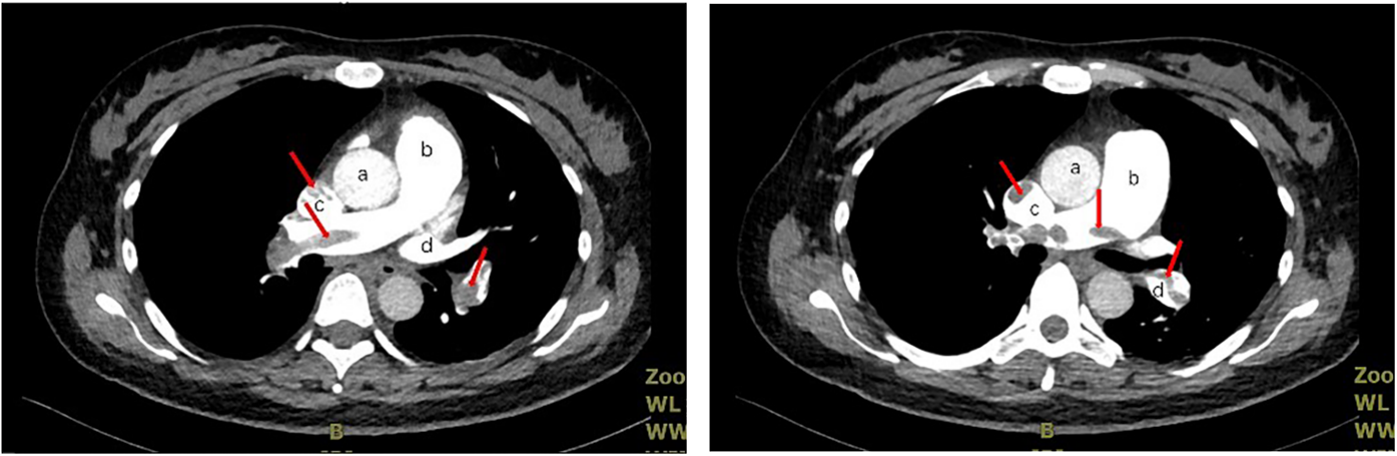
Pulmonary artery CTA image, a, aorta; b, pulmonary artery trunk; c, right pulmonary artery; d, left pulmonary artery (the red arrow indicates multiple embolisms in the left and right pulmonary arteries and pulmonary artery trunks).

**Figure 2:**
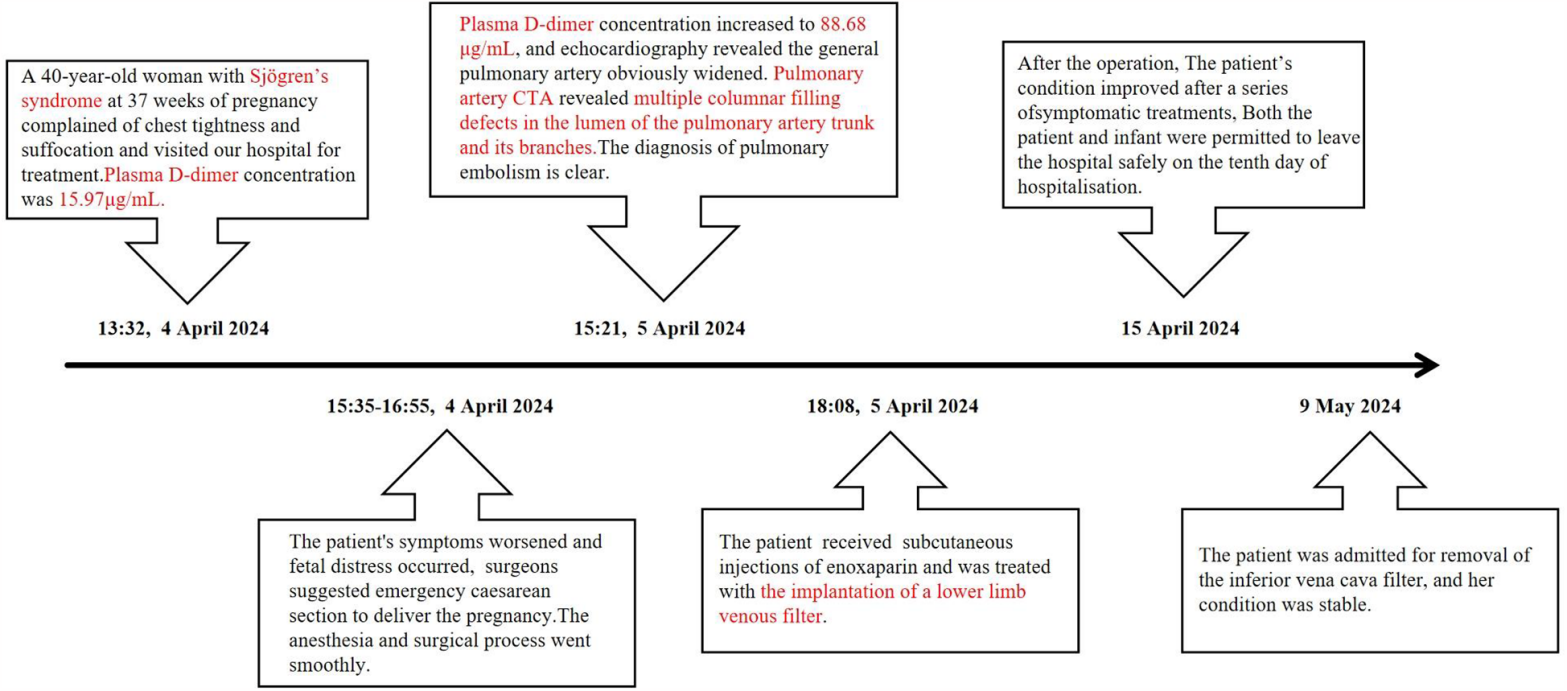
Case timeline.

One day after the operation, although the patient did not complain of discomfort such as chest tightness and breathlessness, the oxygen saturation remained at 95%–96% without oxygen inhalation. The patient received two subcutaneous injections of 60 mg enoxaparin to prevent lower limb thrombosis. The plasma D-dimer concentration increased to 88.68 μg/ml, and echocardiography revealed that the general pulmonary artery was obviously widened, with an estimated pulmonary artery systolic pressure of approximately 37 mmHg and moderate echogenicity in the right pulmonary artery. Pulmonary artery computed tomography angiography (CTA) revealed multiple columnar filling defects in the lumen of the pulmonary artery trunk and its branches (Figure 1). The analysis of the above results supports the clinical diagnosis of multiple PE and right ventricular dysfunction. Multidisciplinary consultations recommend adopting critical care, providing continuous oxygen therapy, maintaining fluid balance, and actively engaging in anticoagulant therapy. In addition, ultrasonography of both lower limbs suggested left popliteal vein thrombosis; thus, the patient was treated with implantation of a lower-limb venous filter. She was transferred to the intensive care unit (ICU) after the operation and received symptomatic treatments such as high-flow oxygen therapy, moxifloxacin for anti-infection, enoxaparin for anticoagulation (enoxaparin 7,500 units subcutaneously every 12 h), gastric protection, and improvement of microcirculation. The patient's symptoms and clinical examination results improved after the above series of therapies in the ICU, and she was transferred to the respiratory department for subsequent treatment 5 days later. Re-examination of lower limb venous ultrasound showed that the venous lumen decreased, blood flow resumed smoothly, plasma D-dimer concentration decreased to 1.69 μm/ml, and there were no obvious abnormalities in others. During this period, the infant was in excellent health and fed milk powder. Both the patient and infant were permitted to leave the hospital safely on the tenth day of hospitalisation. The patient was requested to continue taking oral medication for anticoagulation (rivaroxaban orally, 15 mg twice daily for 2 weeks, then increasing the dosage to 20 mg per dose) and protection of the liver after discharge. The patient was admitted on 9 May 2024 for the removal of the inferior vena cava filter. Her condition was stable, and subsequent treatment included continuing oral rivaroxaban for anticoagulation for 2 months, with regular follow-up appointments (Figure 2).

## Discussion

Epidemiological studies have revealed that the prevalence of SjD, one of the most frequent systemic autoimmune diseases (SAD), is significantly higher in females (up to approximately 95%) than in men (9). There is no evidence suggesting that SjD affects female fertility. Nevertheless, women with SAD are more likely to experience complications during pregnancy than those without corresponding diseases. As one of the target organs during pregnancy, the placentas of patients with SjD may be damaged, causing functional impairment. Maternal antibodies, such as anti-SSA and anti-SSB antinuclear antibodies, can also enter the foetus through the placental barrier and affect intrauterine development, resulting in a high incidence of adverse foetal outcomes (10–12). Pregnancy itself can also affect SjD progression by significantly increasing the production and expression of autoantibodies under the influence of elevated hormone and prolactin levels. Approximately 30% of patients with SjD experience disease progression during pregnancy (13). Fortunately, with the further recognition of SAD and the development of perinatal management, an increasing number of pregnant women with SjD can safely undergo gestation. Notably, manifestations of SjD vary and involve multiple systems. Lung involvement is a common and serious complication, including interstitial lung disease, airway disease, and lymphoproliferative disorders with varying degrees of severity (14).

In contrast, several studies have indicated that patients with SjD, especially those with concomitant interstitial lung disease and anti-SSA antibody positivity, have a higher risk of VTE and PE, which may be related to reduced blood flow velocity and endothelial damage (15, 16). The onset of SjD is accompanied by a significant inflammatory process that produces various cytokines and inflammatory mediators that damage vascular endothelial cells, leading to immune vasculitis and the formation of an inflammatory microenvironment that is conducive to thrombosis (17–19). PE is a clinical and pathophysiological syndrome caused by endogenous or exogenous emboli that block the main trunk or branches of the pulmonary artery, leading to pulmonary circulation disorders. Approximately 75%–90% of PE cases originate from deep vein thrombosis in the lower extremities, and peripartum PE is one of the chief causes of sudden maternal death (20). Pregnant women exhibit an increase in coagulation factors and a decrease in anticoagulant factors, with the fibrinolysis process inhibited. This results in a hypercoagulable state during pregnancy, which may persist for 6–8 weeks postpartum. The vein has a wider diameter and a slower flow velocity during gestation. Physiological compression of the left common iliac vein makes it more prone to thrombosis; thus, researchers agree that peripartum PE mainly originates from thrombosis of the left lower limb. Moreover, caesarean section can cause damage to pelvic tissue and blood vessels, coupled with the stress response of surgery, which can easily activate the body's coagulation system (21).

SjD has diverse clinical manifestations accompanied by multiple organ injuries, and pregnancy can increase the complexity of the illness. Therefore, perioperative anaesthetic management of SjD combined with pregnancy remains a challenge. In this case, the patient had been diagnosed with SjD in the previous prenatal examination, mainly due to respiratory and circulatory symptoms, including chest tightness, shortness of breath, and palpitation, which in turn required an emergency caesarean section to deliver the pregnancy. First, adequate preoperative assessment of patients according to their general condition is crucial, focusing on symptoms and simultaneously discriminating against other fatal complications at the same time. The detection of anti-SSA, ANA, and anti-Ro52 antibodies on admission, combined with typical related symptoms (dry mouth, dry eyes, and Raynaud phenomenon), confirmed the diagnosis of SjD (9). The available literature has identified that the cardiovascular system is a target of SjD and that all parts of the heart can be affected, mainly characterised by pulmonary arterial hypertension, pericarditis, and cardiac arrhythmias (22). With this in mind, B-type natriuretic peptide, sensitive troponin I, creatine kinase isoenzymes, and electrocardiograms were detected and observed, and no apparent abnormalities were found. The patient's preoperative plasma D-dimer levels increased abnormally. D-dimer is a soluble fibrin degradation biomarker that can be measured in whole blood or plasma. The fibrinolytic system produces it through the orderly decomposition of thrombi and has been extensively investigated for the diagnosis of VTE or PE. In terms of the outcome, the increase in D-dimer levels in this case indicated the subsequent occurrence of PE. However, there are some limitations to D-dimer testing for the diagnosis of the above diseases: the specificity of detection decreases with pregnancy, cancer, recent surgery, or trauma, and D-dimer levels increase with gestational age and complicated pregnancies; therefore, combining the results of imaging examinations may increase the diagnostic accuracy (23).

It is more likely that deep vein thrombosis (DVT) and PE were present before surgery, and we chose combined spinal-epidural anaesthesia after eliminating the relative taboos. Compared to general anaesthesia, intraspinal anaesthesia inhibits the sympathetic nervous system, dilates blood vessels below the blocking plane, and accelerates blood flow in the lower limb blood vessels (24). Intraspinal anaesthesia also has a positive effect on blood rheological properties, significantly reducing red blood cell viscosity and effectively inhibiting blood clots (25). Local anaesthetics, including ropivacaine, can inhibit coagulation by preventing platelet aggregation and particle release (26). Although the arterial blood gas analysis before anaesthesia was normal, we found that the blood oozing out during intraspinal anaesthesia was dark black, adding to the situation that she had sustained low oxygen saturation and ETCO_2_ pressure with symptoms of cyanosis of the lips, chest tightness, and suffocation. There are reasons to doubt that the patient may have experienced PE during surgery. Fortunately, continuous intraoperative oxygen therapy and accurate fluid management are crucial to ensure a smooth operative finish, and both the mother and baby were doing well after the operation.

The D-dimer concentration continued to rise after the caesarean section for some time, and an accurate clinical diagnosis of PE and DVT was made, ultimately relying on pulmonary artery CTA and ultrasound. Subsequently, the patient underwent all interventions in sequence, including lower-limb venous filter implantation, anticoagulation, oxygen therapy, and other symptomatic treatment measures, and was finally discharged from the hospital on the tenth day of hospitalisation.

The outcomes of this case were satisfactory. Several aspects of the perioperative period are worth discussing and further improving. First, when faced with a high degree of suspected PE with anoxic signs, pectoralgia, or other conditions, related examinations, including bedside cardiac and lower limb ultrasonography and pulmonary artery CTA, should be performed to immediately clarify the diagnosis before surgery, which is available for subsequent haemodynamic and respiratory support therapy. Simultaneously, anticoagulants or thrombolytic therapy should be administered as early as possible. Heparin is a commonly used anticoagulant in clinical practice, and the injection of heparin after caesarean section is mainly used to prevent DVT in the lower limbs. Guidelines recommend administering anticoagulant drugs 6–12 h after caesarean section to reduce the risk of perinatal bleeding in patients with PE (27). Thrombolytic drugs can directly or indirectly convert fibrinogen into fibrinolytic enzymes, rapidly degrade fibrin, and dissolve blood clots. Thrombolytic therapy with urokinase or recombinant tissue-type plasminogen activator combined with anticoagulant therapy has a positive effect on patients with acute PE. In a study of 28 cases of thrombolysis during pregnancy, Leonhardt et al. found that the effective rate was 90%, the foetal mortality rate after thrombolysis treatment was 23% (including three cases of induced abortion), and no significant sequelae were found in surviving newborns after delivery (28). Therefore, systemic or catheter-directed thrombolysis may have been a good treatment option for this patient. Second, in addition to routine monitoring such as electrocardiography, non-invasive blood pressure, pulse oximetry, and body temperature, invasive measurement of continuous arterial pressure should be established to monitor circulatory systemic situations in real time and obtain blood samples for arterial blood gas analysis. General anaesthesia may be a better option for PE patients with a confirmed diagnosis and unstable haemodynamics or for those who have already received anticoagulant treatment. Finally, the epidural catheter was removed immediately after surgery; however, considering that the patient would soon receive anticoagulant or thrombolytic therapy, it may be more favourable to postpone catheter removal contingent on different medication regimens.

## Conclusion

Patients with SjD are at a high risk of developing PE and VTE during pregnancy. The key to dealing with such cases is to quickly identify the early onset of signs, definitively diagnose them, and provide direct treatment with the help of imaging examinations. Therefore, high-quality perioperative anaesthesia management, as typified by appropriate oxygen therapy and volume management, is helpful for improving patient prognosis.

## Data Availability

The raw data supporting the conclusions of this article will be made available by the authors, without undue reservation.
